# Consideration of Sustainability When Approving Human Medical Research—A Scoping Review

**DOI:** 10.1007/s11673-024-10365-9

**Published:** 2024-07-25

**Authors:** Tony Skapetis, Bernadette Nicholl, Kellie Hansen

**Affiliations:** 1 NSW Health, Western Sydney Local Health District, Westmead, NSW Australia; 2 The University of Sydney School of Dentistry, Faculty of Medicine and Health, The University of Sydney, Sydney, NSW Australia

**Keywords:** Research sustainability, Ethical considerations, Health research, Ethics committees, Institutional review boards

## Abstract

This article attempts to highlight the importance of including research sustainability as imperative when assessing human medical research in terms of ethical principles. Using a scoping review of recent literature, the complexity of research sustainability is highlighted with key themes and concepts surrounding this important topic being recognized and discussed. An overall paucity of guidance documents was identified and recommendations have been made to practically address this deficiency. An example of a research sustainability evaluation tool which is currently being piloted has been provided for possible adaptation and use by Ethics Committees and Institutional Review Boards to bolster the concept and inclusion of sustainability during the research approval process.

## Background

The background has been divided into several sub-headings to help the reader better navigate the complexities surrounding research sustainability.

### Defining Research Sustainability and the Purpose of This Paper

The term “sustainability” is used across a broad conversation encompassing social, environmental, and economic constructs (Browne 2022). There are however various definitions of sustainability as identified by a literature review by Moore (Moore, et al. 2017) and more recent reviews explain how the definitions around sustainability are constantly developing and emerging (Ruggerio 2021). In another paper, sustainability is defined as “meet(ing) the needs of the present without compromising the ability of future generations to meet their own needs” (Gabrielle and Cristina 2023, 429). It should be noted that this latter definition is considered “rather vague” and has been the source of considerable contention (Ravago, et al. 2015). The accepted definitions of sustainability generally describe three pillars, environmental, economic, and social. That is, processes that do not cause irreversible change to the environment, that are economically viable, and benefit society (Rodriguez, et al. 2002). Research sustainability describes research that is designed and delivered in a way that minimizes the impact and enhances the benefits environmentally, socially, culturally, and economically to the communities improved by that research (Charles Sturt University 2019).

Helping both guide and enhance research ethical review, the National Health and Medical Research Council (NHMRC) in partnership with a consortium of Australian Universities, has developed a comprehensive guidance document titled the “National Statement on Ethical Conduct in Human Research 2023.” The latest version of this document was published in mid-2023 and is the main guidance document used by HREC’s in Australia and took effect in January 2024 (NHMRC 2023). The purpose of this paper is to provide some guidance for HREC’s both in Australia and internationally, beyond what is available in the National Statement, in terms of inclusion of sustainability within the research review process. This was aided through a scoping review of the current literature with a general focus pertaining more towards a reduction in Carbon footprint as this has been more widely reported with association to medical research. However, other aspects of research sustainability such as research bias and wastage, possible exemptions as well as pollution are among those discussed.

### Economics of Research Sustainability

Globally, healthcare is moving toward for-profit operational models. If instead, healthcare and health research moved to ethical economics and promoted humanistic healthcare, this would contribute to a reduced carbon footprint, reduction of the harm caused to communities, particularly those in developing countries who do not benefit from clinical trials but rather experience harm from the climate change they contribute to (Samuel 2023).

In 2015, the 2030 Agenda for Sustainable Development was adopted by United Nations Member States. This document outlined seventeen Sustainable Development Goals (SDG) to be achieved by 2030. These all relate to improving lives and protecting the environment and all depend on strong economic modelling. These elements similarly underpin the model of Doughnut Economics which is being implemented by many businesses and communities and is a good fit for research sustainability (Raworth 2017).

### Exemptions within Sustainability

Despite all the research, evidence, and strategies, there are circumstances when aspects of research sustainability may have a diminished priority and may need possible exemption. An example of this occurred during the COVID pandemic. The latter had a short-term positive impact on CO2 emissions and air quality with fewer cars on the road, planes in the air, and most factories temporarily closed. However, the large number of single-use personal protective equipment (PPE) required, estimated globally to be 129 billion facemasks and 65 billion gloves each month, the haphazard disposal of these, and the significant volume of hospital waste generated, raised important questions about safe waste management, possible pollution, and eliminated gains within the broader construct of sustainability (NSW Health 2020).

### Ethical Considerations

Within the global medical research landscape, Australia is perceived as a world stakeholder in medical research and has invested heavily with billions of dollars being allocated through various sources including government contributions such as the Medical Research Future Fund (Department of Health and Aged Care 2023) as well as industry sponsored investment. Most of this research undergoes some form of ethical review via Human Research Ethics Committees (HREC).

Western Sydney Local Health District (WSLHD) is a large state-based (New South Wales) provider of health-related services to 1.5 million of the Sydney population. Additionally, it hosts a substantial medical research and governance portfolio including a large HREC which has acknowledged the imperative of incorporating the concept of research sustainability within its decision-making process.

Ethical review of human research is generally seen to have a positive effect on research outcomes but not without criticism which often relates to delays in approval of studies which can be viewed as obstructionist (Martinez 2023). Nevertheless, this paper attempts to advocate that Human Ethics and Institutional Review Boards should include as an additional consideration the concepts of sustainability within their remit when approving research.

### Research Sustainability and Quality

Although sustainability is always present in the background of contemporary scholarship and research, literature which examines the interconnections, discourses, and dimensions of sustainability in research is still being developed and requires further investigation. There is however agreement that sustainability and ethics bisect in terms of societal values, morals, and norms (Walsh et al. 2021).

The quality of each project should be considered from the ethical and research sustainability perspective to ensure robust, efficiently run projects, which will also show improved health outcomes. The research sustainability of projects can be improved and assessed using the 4Rs Reduce, Reuse, Recycle, Replace. Considering the volume of research projects undertaken globally, single ethics review, approval across multiple sites, using platform or umbrella designs, and creating tiered review pathways based on risk profiles are all steps towards sustainable research (Glasziou, et al. 2021).

All medical research has a carbon footprint even if the results are not published. Human research relies heavily on data and digital infrastructure to store, manage, and analyse the data generated by research. Digital infrastructure has a rapidly expanding impact on carbon emissions worldwide. Artificial Intelligence (AI) is doubling its thirst for digital data every three to four months, and is therefore outpacing efficiency, while at the same time increasing waste, toxins, and environmental damage. The digital sector is calculated to contribute between 2.1 per cent and 3.9 per cent of global carbon emissions (Samuel 2023). Medical research is a small portion of all digital technology, but it is also the fastest-growing sector with increasingly significant impact over time (NIHR 2019). The carbon footprint of healthcare in Australia for example, represents approximately 7 per cent of the country’s total, which is significant (NSW Health 2022).

Reducing the carbon footprint of healthcare relies on evidence-based strategies developed from sustainably conducted research. It has been well documented that pollution from solid waste and emissions negatively effects both acute and chronic health outcomes. This has led the same authors to suggest that the way forward is to apply Health System Science frameworks to sustainability stewardship. This includes the core domains of structure/process, policy/economics, information/technology, determinants of health, value, and system improvements (Sood and Teherani 2022). Other authors suggest that research sustainability should be viewed in terms of scientific quality, social value (including environmental), respect for persons, communities and the environment, justice and favourable benefit to risk ratios (Gabrielle and Cristina 2023). Walsh and colleagues (2021) on the other hand, considers sustainability in terms of ontology, epistemology, and the ethical stance of what is morally good, bad, right, and wrong. These authors identify three key relational concepts to ethics sustainability, namely, ecocentrism (ecology), biocentrism (biology), and anthropocentrism (humanity).

## Literature Search Strategy

A well-structured search strategy was employed to identify relevant literature, by an experienced medical librarian who was also a co-author. Two databases were employed for the search, namely Medline and Embase (via Ovid) using the dates 1996 to December 18, 2023. The Medline search terms used were “ethics committees” (research and clinical), “sustainability” (checklist, guideline, framework, and model) and “clinical trial.” The search terms “sustainability” (checklist, guideline, framework, and model) and “clinical trials” were employed for the Embase search. Additionally, the reference lists of identified articles were used to supplement the electronic search. The initial date range for Medline and Embase is set at 1996 by default and this was adopted for this search. It also became evident upon reviewing the search results, that the articles most relevant to this topic were predominantly published within the last decade.

## Findings of the Scoping Review

The review identified a diversity of research sustainability themes among the literature which has been grouped under the following convenient headings.

### Publication Bias as Unnecessary Wastage

Waste in medical research has historically been defined in terms of design flaws, non-publication, and inadequate reporting. It is estimated this renders over 85 per cent of research a wasted effort and equates to over 100 billion dollars of avoidable research waste globally every year (Institute for Evidence-Based Healthcare 2023). If waste in medical research is viewed through a sustainability lens to include such constructs as resource consumption, carbon emissions, and possible pollution, the associated impact increases significantly. It is estimated that approximately 85 per cent of all health research is being avoidably “wasted” and up to 50 per cent of registered clinical trials are never published in full (Chalmers and Glasziou 2009).

### Carbon Footprint Reduction

Authors have reported that in 2022 there were over 400,000 clinical trials registered under ClinicalTrials.gov without the inclusion of environmental impact as part of regulatory frameworks (Billiones 2022). Clinical trials within the USA health system are responsible for 8.5 per cent of total annual carbon emissions and it is estimated that 13.1 kg of waste is generated per hospital bed per day (Sood and Teherani 2022). In other studies, the global environmental impact of clinical trials has been equated to an equivalency of almost one third of the annual carbon emission of Bangladesh’s 163 million population (Gabrielle and Cristina 2023).

Most Governments have policies for reducing their carbon footprint across all sectors, including their health services, with few addressing the carbon footprint of medical research. In a cross-sectional survey of Clinical Trial Networks involving fifteen countries from four continents, half of the networks reported that few if any research projects considered carbon footprint reduction and the remaining half where not familiar with this concept. The same study reported that 60 per cent of respondents reported that Institutional Review Boards (IRB) and HREC’s did not consider carbon footprint reduction and the remaining 40 per cent were unsure (Hoffmann et al. 2023). Similarly, another author reported that one fifth of clinical trials published in six medical journals were primarily for marketing purposes (Adshead, et al. 2021) and therefore could be viewed as wasted research that could contribute to the Carbon footprint.

In the United Kingdom, the methods used to calculate the carbon footprint of the National Health Service (NHS) are the most comprehensive to date (NSW Health 2022). While they are extensive, they are not inclusive of medical research. The impact of medical research on the overall carbon footprint and the resulting harm to the health of the community has resulted in the formation of the National Institute for Health and Care Research (NIHR) Carbon Reduction Guidelines. In the United Kingdom the Climate Change Act has set targets for all sectors. The commitment made by the NHS to meet those targets also includes medical research (NIHR 2019).

### Guidance Towards More Sustainable Research

Internationally, tools have been developed for researchers such as the care pathway carbon calculator and a clinical trials guidance tool for appraising sustainability, by the Sustainable Health Coalition (Hoffmann et al. 2023). Despite such tools, the guiding documents used by ethics committees are often largely based on a risk–benefit assessment. Currently, environmental risks are not defined as part of this assessment (Billiones 2022).

In the Australian research landscape, the National Statement on Ethical Conduct in Human Research (NHMRC 2023) outlines the core principles of respect, beneficence, merit and integrity, and fairness. These fundamental principles apply equally to the concept of research sustainability. The Australian Institute of Aboriginal and Torres Strait islander Studies states research should be environmentally, culturally, socially, and economically sustainable, advocating no harm to land, waterways, climate change, and people (Australian Institute of Aboriginal and Torres Strait Islander Studies 2020).

To improve research sustainability, in addition to good project design, systemic and operational solutions are necessary. Systemic impacts that can be managed in health research include reducing waste and pollution, including greenhouse gas emissions. Engaging in sustainable procurement practices and direct initiatives to reduce resource consumption including reusing already collected data, shared reference libraries, avoid printing and copying, and collaborating with colleagues. Operational impacts can include improving energy efficiency and reducing overall energy use by considering transport options, environmental considerations in work spaces and new building projects, reuse of equipment where possible, online meetings and study visits (Charles Sturt University 2019).

Additionally, similar strategies were proposed by Glasziou et al. (Glasziou et al. 2021) which included reducing, re-using, or re-adapting previous submissions, recycling of generic protocols, and introducing more efficient review processes. When condensed and synthesized, together with previously mentioned themes from this scoping review, two common constructs were preferenced by the authors using a pragmatist approach. Namely, the concepts of “Data Maximization” and “Research Efficiency,” which can be used more broadly to help evaluate research sustainability from an ethical perspective. The rationale for this simplification is not to make such an evaluation exhaustive but rather to make this easily applicable for researchers and HRECs to adopt, thereby helping guide the compass of research sustainability in the right direction.

Given that limited guidance is provided by the aforementioned literature for researchers and ethics committees on how to understand, demonstrate, or assess research sustainability of projects under review. The development of a companion tool for ethics committees with which to consider a research proposal could potentially further assist HREC’s in Australia and IRB’s more broadly, in evaluating their sustainability credentials.

**Fig. 1:**
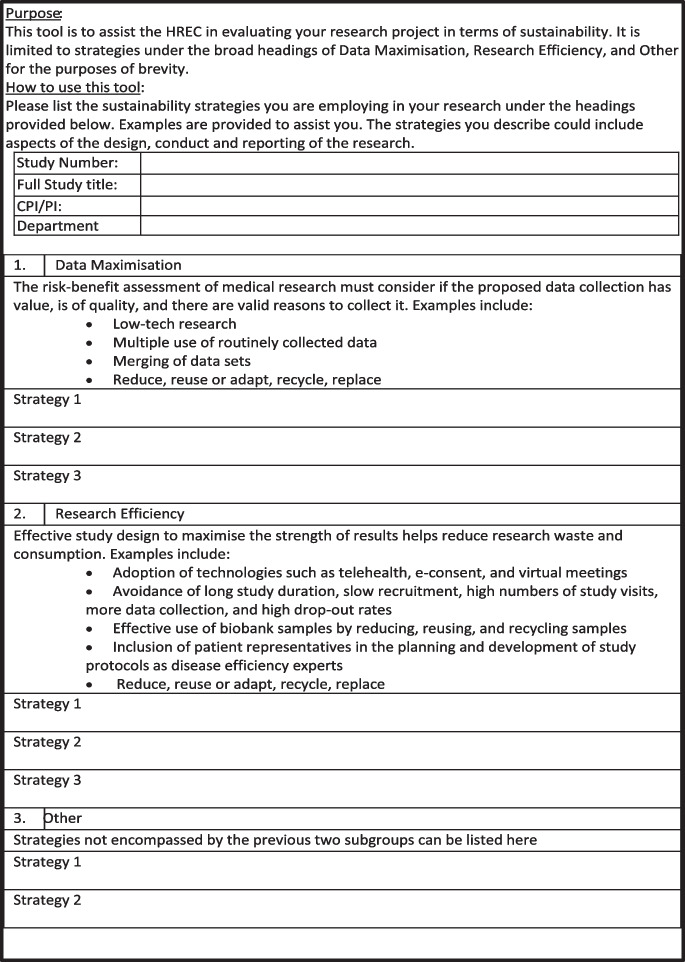
Research Sustainability Tool

To that end a simple tool (Fig. 1) is being piloted for six months in WSLHD. Researchers undertaking Greater than Low Risk Research are required to submit this tool with their application for review by the HREC. There is however an argument to be made that such a tool could also be used prior to any ethical review and during the planning stage of any research. As this is a new concept in human research, at least within Australia, the pilot is looking to “plant a seed” and encourage the inclusion of research sustainability as a standard part of the project planning stage. Further review of the literature and an evaluation of the tool and its impact, will need to be undertaken at the end of the pilot. The tool will be revised and a guidance document to support use of the tool will be developed based on the outcome of the evaluation.

## Strengths

The scoping review search methodology was conducted using the expertise of an experienced hospital librarian. This is the first documented application of sustainability constructs when considering human research ethical review in Australia.

## Limitations

The search methodology was limited to a scoping review of the literature rather than being systematic. Themes relating to research ethics sustainability may have been missed and overly simplified. The tool was developed largely for use by an Australian HREC and may not necessarily translate directly to other HREC’s and IRB’s in other countries and jurisdictions.

## Conclusion

A scoping review surrounding human research ethics sustainability was conducted. It identified both the complexities associated with this topic as well as the multiple dimensions relating to research sustainability which were condensed under the headings of “Data Maximization” and “Research Efficiency.” These two pragmatically derived constructs were subsequently used in the genesis of a sustainability tool which is currently being piloted by a major Australian HREC and may be adaptable for use by other HRECs and IRBs more broadly.

The literature review findings were grouped under the subheadings of publication bias and wastage, carbon footprint reduction and a guidance section for more sustainable research, in order to provide an additional scaffold through which other HRECs could adopt research sustainability within their approval deliberation processes.
